# LPS Response Is Impaired by Urban Fine Particulate Matter

**DOI:** 10.3390/ijms23073913

**Published:** 2022-04-01

**Authors:** Natália de Souza Xavier Costa, Gabriel Ribeiro Júnior, Adair Aparecida dos Santos Alemany, Luciano Belotti, Marcela Frota Cavalcante, Susan Ribeiro, Mariana Matera Veras, Esper Georges Kallás, Paulo Hilário Nascimento Saldiva, Marisa Dolhnikoff, Luiz Fernando Ferraz da Silva

**Affiliations:** 1 Departamento de Patologia, LIM-05, Faculdade de Medicina da Universidade de São Paulo, São Paulo 01246-903, Brazil; gabriel_ribe@usp.br (G.R.J.); adair.alemany@gmail.com (A.A.d.S.A.); lucianobelotti@yahoo.com.br (L.B.); verasine@usp.br (M.M.V.); pepino@usp.br (P.H.N.S.); maridol@usp.br (M.D.); burns@usp.br (L.F.F.d.S.); 2 Departamento de Análises Clínicas e Toxicológicas, Faculdade de Ciências Farmacêuticas da Universidade de São Paulo, São Paulo 05508-000, Brazil; marcela.frotac@gmail.com; 3 Laboratório de Imunologia Clínica E Alergia (LIM60), Faculdade de Medicina da Universidade de São Paulo, São Paulo 01246-903, Brazil; susanprm@gmail.com (S.R.); esper.kallas@gmail.com (E.G.K.); 4 PATRU, Department of Pathology, Emory University, Atlanta, GA 30322, USA

**Keywords:** air pollution, acute lung injury, lipopolysaccharides, inflammatory response

## Abstract

Fine particulate matter (PM_2.5_) is a complex mixture of components with diverse chemical and physical characteristics associated with increased respiratory and cardiovascular diseases mortality. Our study aimed to investigate the effects of exposure to concentrated PM_2.5_ on LPS-induced lung injury onset. *BALB/c* male mice were exposed to either filtered air or ambient fine PM_2.5_ in an ambient particle concentrator for 5 weeks. Then, an acute lung injury was induced with nebulized LPS. The animals were euthanized 24 h after the nebulization to either LPS or saline. Inflammatory cells and cytokines (IL-1β, IL-4, IL-5, IL-6, IL-10, IL-17, TNF) were assessed in the blood, bronchoalveolar lavage fluid (BALF), and lung tissue. In addition, lung morphology was assessed by stereological methods. Our results showed that the PM+LPS group showed histological evidence of injury, leukocytosis with increased neutrophils and macrophages, and a mixed inflammatory response profile, with increased KC, IL-6, IL-1β, IL-4, and IL-17. Our analysis shows that there is an interaction between the LPS nebulization and PM_2.5_ exposure, differently modulating the inflammatory response, with a distinct response pattern as compared to LPS or PM_2.5_ exposure alone. Further studies are required to explain the mechanism of immune modulation caused by PM_2.5_ exposure.

## 1. Introduction

The burden of diseases associated with urban air pollution exposure is large; it especially increases morbidity and mortality from non-communicable cardiovascular and respiratory diseases [1]. Particulate matter (PM) is a complex mixture with diverse chemical and physical characteristic components. To date, strong evidence shows a correlation between fine PM_2.5_ (particulate matter with an aerodynamic diameter <2.5 μm) exposure and all-cause mortality, such as acute lower respiratory infections, chronic obstructive pulmonary disease, ischemic heart disease, lung cancer, and stroke [2].

The literature abounds with studies showing the effects of individuals chronically exposed to air pollution on their lung health. In humans, chronic exposure to air pollution is associated with decreased lung function [3], increased risk of developing acute respiratory distress syndrome [4], chronic asthma, and pulmonary insufficiency [5], and inflammation has been implicated as the key mechanism of PM-mediated health problems. In addition, PM_2.5_ exposure can modulate different inflammatory pathways causing a Th1/Th2 and Th17/Treg imbalance [6,7].

The lipopolysaccharide (LPS) is the most abundant component of the Gram-negative bacteria outer membrane and it is composed of a hydrophilic polysaccharide and a highly conserved hydrophobic component referred to as lipid A, which is responsible for the major bioactivity of endotoxin and is recognized by the host immune cells [8]. The asymmetric distribution of the LPS on the surface of the outer membrane is an effective barrier against small, hydrophobic molecules, including many antibiotics [9]. Rapid growth and cell division depend on the bacteria's capacity to synthesize and export LPS efficiently and in large amounts [10].

LPS also plays a crucial role in bacteria–host interactions by modulating responses by the host immune system, triggering a macrophage-mediated inflammation. Nevertheless, exposure to repeated doses of LPS can lead to tolerance. The LPS tolerance has been described in several species, including humans, suggesting that tolerance may be a protective adaptation to LPS exposure [11].

Previous studies with animal models of LPS-induced acute lung injury and exposure to different types of PM_2.5_ showed aggravation of LPS-related lung injury, neutrophil recruitment, increased oxidative stress, and expression of pro-inflammatory cytokines [12,13]. In most of these experimental studies, the PM_2.5_ exposure is either concomitant with the LPS exposure or only a few days before the LPS instillation [14]. The effects of chronic exposure to PM_2.5_ on the onset and progression of LPS-induced injury are not known. Therefore, this study aims to evaluate how the sub-chronic exposure to PM_2.5_ can alter the inflammatory response of the LPS-induced acute lung injury onset. To our knowledge, this is the first study to assess in vivo the effects of a sub-chronic exposure to “real-world” PM_2.5_ followed by an LPS challenge.

## 2. Results

### 2.1. Exposure and PM_2.5_ Characterization

According to Andrade et al. [15] and de Miranda et al. [16], the air pollution at the exposure site was predominantly characterized as emission from vehicular sources. The exposure took place during the dry season and the mean temperature and humidity during the exposure period were 24.3 °C and 55.8%, respectively. The mean PM_2.5_ dose was 1219.1 ± 616.7 µg·m^−3^ and the associated 24 h mean PM_2.5_ exposure was 50.8 µg·m^−3^.

Characterization of the PM_2.5_ collected in filters was previously reported elsewhere. Lopes et al. [17] reported the concentration of PM_2.5_, its black carbon (BC), and metal trace content from the collected filters. Yoshizaki et al. [18] reported the polycyclic aromatic hydrocarbon content, and Costa et al. [19] reported the endotoxin content.

### 2.2. Histopathological Description

**Figure 1:**
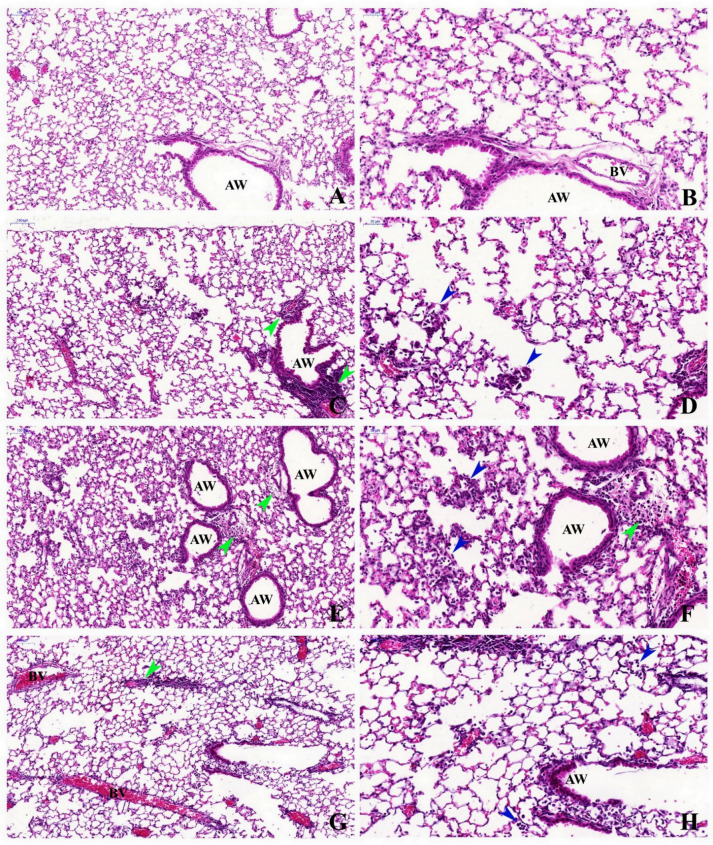
Representative photomicrographs of lung tissue (H&E staining). Control group: (**A**) 10× and (**B**) 20×, thin alveolar septa and no significant inflammation. PM group: (**C**) 10× and (**D**) 20×, inflammatory infiltrate alongside the bronchovascular bundle and mild presence of inflammatory cells in the alveolar space. LPS group: (**E**) 10× and (**F**) 20×, septal thickening and intense inflammatory infiltrate in the alveolar space and adjacent to the airways and blood vessels, with a predominance of polymorphonuclear cells. PM+LPS group: (**G**) 10× and (**H**) 20×, septal thickening, perivascular and peribronchial inflammatory infiltrates, and presence of inflammatory cells in the alveolar space. AW: Airway. BV: Blood Vessel. Green Arrowhead: inflammatory cell infiltration in the peribronchial and perivascular areas. Blue Arrowhead: interalveolar inflammatory cells. 10×: scale bar = 100 μm and 20×: scale bar = 50 μm.

The histopathological evaluation of the lung tissue showed that the control group had a normal appearance, with no significant inflammation nor septal thickening (Figure 1A,B). The PM group presented inflammatory infiltrate alongside the bronchovascular bundle and mild infiltration of inflammatory cells in the alveolar space, with a predominance of mononuclear cells (Figure 1C,D). The LPS group showed important inflammatory infiltrate in the alveolar space and adjacent to the airways and blood vessels, with a predominance of polymorphonuclear cells. There was also septal thickening, alveolar air spaces widening with an irregular distribution, and focal areas of alveolar hemorrhage (Figure 1E,F). In the PM+LPS group, the lung tissue presented perivascular and peribronchial inflammatory infiltrates, presence of inflammatory cells in the alveolar space, septal thickening, and alveolar air spaces widening with an irregular distribution (Figure 1G,H). The semi-quantitative analysis showed a significant increase in the peribronchial inflammation score in the LPS and PM+LPS groups compared to the control (*p* ≤ 0.0001 and *p* = 0.001, respectively) and PM (*p* = 0.01 and *p* = 0.03) groups. The alveolar inflammatory infiltrate score was also higher in the LPS group compared to the control (*p* = 0.001) and PM (*p* = 0.001) groups and tended to be higher in the PM+LPS group compared to the control (*p* = 0.06) and PM (*p* = 0.06) groups. The peribronchial inflammation score and alveolar inflammatory infiltrate score were both influenced by LPS nebulization (*p* ≤ 0.0001, for both variables) (Table 1).

### 2.3. Stereological Analysis

The body weight at the beginning of the exposure period was not different among the groups. The final body weight was decreased in the LPS and PM+LPS groups compared to the control (*p* = 0.022 and *p* = 0.008, respectively) and PM (*p* ≤ 0.0001 and *p* ≤ 0.0001, respectively) groups. The two-way ANOVA analyses showed that the body weight was influenced by the LPS nebulization (*p* ≤ 0.0001). The total lung volume was not statistically different among the groups. The lung volume per body weight ratio was increased in the PM+LPS group compared to the control (*p* = 0.04), also influenced by the LPS nebulization (*p* = 0.02).

The volume density and total volume of the lung parenchyma were not different among the groups. The septa volume density was increased in the PM+LPS group compared to the control (*p* = 0.049), due to the LPS nebulization (*p* = 0.031), and the alveolar air space volume density was decreased in the PM+LPS group compared to the LPS group (*p* = 0.049), as a consequence of the PM_2.5_ exposure (*p* = 0.01). Furthermore, the total septa volume was increased in the LPS group compared to the control (*p* = 0.024) group and the alveolar total volume was decreased in the PM+LPS group compared to the LPS (*p* = 0.02) group, both due to the interaction between LPS nebulization and PM_2.5_ exposure (*p* = 0.002 and *p* = 0.004, respectively).

The volume density and total volume of the non-parenchyma structures were not different among the groups.

The septa surface density was decreased in the LPS (*p* = 0.035) and PM+LPS (*p* = 0.018) groups compared to the control due to the LPS nebulization (*p* = 0.01); however, there was no difference in the septa total surface area among the groups.

The septum arithmetic mean thickness was increased in the LPS (*p* = 0.018) and PM+LPS (*p* = 0.023) groups compared to the control, influenced by the LPS nebulization (*p* = 0.004).

All the stereological data are summarized in Table 1.

### 2.4. Inflammatory Cells Assessment

The data from the red blood cell count, fibrinogen, and platelet quantification are presented in Supplementary Table S1.

**Figure 2:**
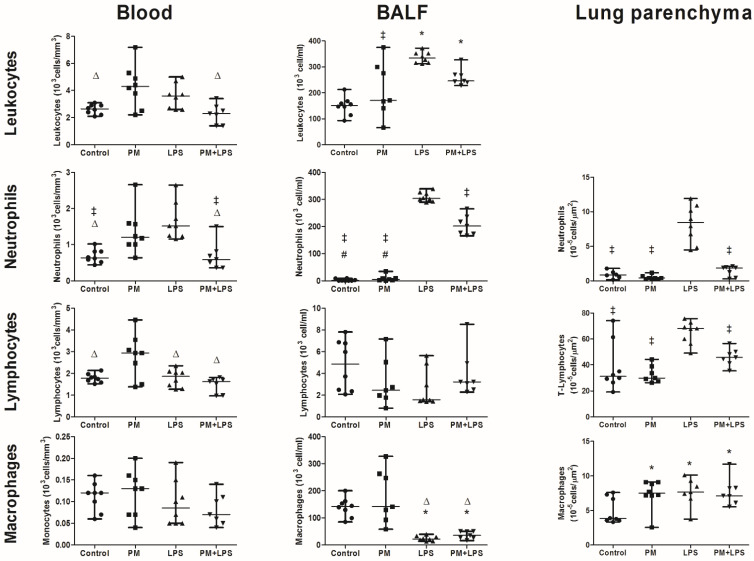
Graphical representation of inflammatory cells assessed in the blood (10^3^ cells/mm^3^), BALF (10^4^ cells/mL), and lung parenchyma (10^−5^ cells/μm^2^). Each point represents one animal and bars show the median. * *p* < 0.05 compared to the control group. ^‡^ *p* < 0.05 compared to the LPS group. ∆ *p* < 0.05 compared to the PM group. # *p* < 0.05 compared to the PM+LPS group.

The circulating leukocytes were increased in the PM group compared to the control (*p* = 0.012) and PM+LPS (*p* = 0.003) groups, as a result of the interaction between the LPS nebulization and PM_2.5_ exposure (*p* ≤ 0.0001). The BALF leukocytes were increased in the LPS group compared to the control (*p* ≤ 0.0001) and PM (*p* = 0.002) groups. It was also increased in the PM+LPS group compared to the control (*p* = 0.005) group. The BALF leukocytes outcome was influenced by the LPS nebulization (*p* ≤ 0.0001) and by the interaction between the LPS nebulization and PM_2.5_ exposure (*p* = 0.003) (Figure 2).

The circulating neutrophils were increased in the LPS group compared to the control (*p* = 0.001) and PM+LPS (*p* = 0.002) group. In addition, the PM group also displayed increased neutrophils compared to the control (*p* = 0.03) and PM+LPS (*p* = 0.039) groups, influenced by the interaction between the LPS nebulization and PM_2.5_ exposure (*p* ≤ 0.0001). The BALF neutrophils were increased in the LPS and PM+LPS groups compared to both the control (*p* ≤ 0.0001 and *p* ≤ 0.0001, respectively) and PM (*p* ≤ 0.0001 and *p* ≤ 0.0001, respectively) groups, because of the LPS nebulization (*p* ≤ 0.0001), PM_2.5_ exposure (*p* ≤ 0.0001), and their interaction (*p* ≤ 0.0001). MPO+ neutrophils, assessed in the lung parenchyma, were increased in the LPS group compared to the control (*p* ≤ 0.0001), PM (*p* ≤ 0.0001), and PM+LPS (*p* ≤ 0.0001) groups, also influenced by the LPS nebulization (*p* ≤ 0.0001), PM_2.5_ exposure (*p* ≤ 0.0001), and their interaction (*p* ≤ 0.0001) (Figure 2).

The circulating lymphocytes were increased in the PM group compared to the control (*p* = 0.011), LPS (*p* = 0.01), and PM+LPS (*p* = 0.001) groups, as a consequence of the PM_2.5_ exposure (*p* = 0.005). The BALF lymphocytes were not different between the groups. The proportion of CD3+ lymphocytes in the lung parenchyma was increased in the LPS group compared to the control (*p* = 0.001), PM (*p* ≤ 0.0001), and PM+LPS (*p* = 0.028) groups, influenced by LPS nebulization (*p* ≤ 0.0001) and PM_2.5_ exposure (*p* = 0.009) (Figure 2).

There was no statistical difference in the circulating monocytes among the groups. The BALF macrophages were decreased in the LPS and PM+LPS groups compared to the control (*p* = 0.004 and *p* = 0.014, respectively) and PM (*p* = 0.001 and *p* = 0.003, respectively) groups, as a result of the LPS nebulization (*p* ≤ 0.0001). The proportion of MAC2+ macrophages present in the lung parenchyma was increased in the LPS (*p* = 0.008), PM (*p* = 0.009), and PM+LPS (*p* = 0.017) groups compared to the control group, influenced by the LPS nebulization (*p* = 0.026), PM_2.5_ exposure (*p* = 0.043), and their interaction (*p* = 0.011).

**Figure 3:**
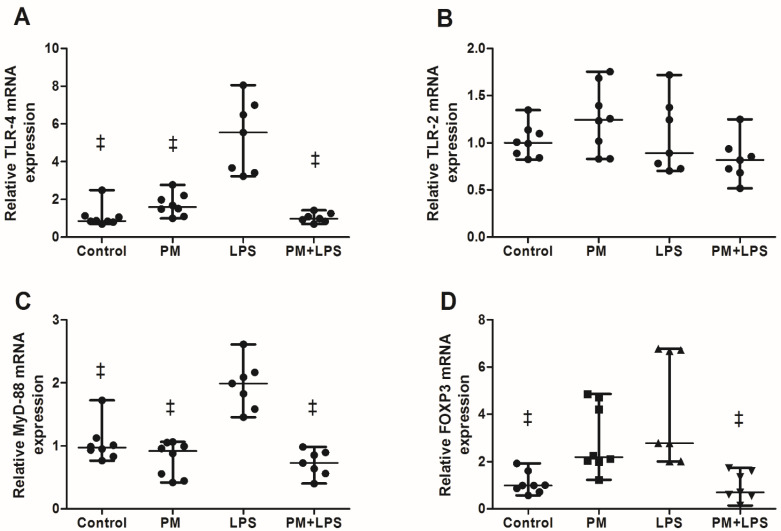
Graphical representation of (**A**) relative TLR-4 mRNA expression, (**B**) relative TLR-2 mRNA expression, (**C**) relative MyD-88 mRNA expression, and (**D**) relative FOXP3 mRNA expression assessed in the lung tissue. Each point represents one animal and bars show the median. ^‡^ *p* < 0.05 compared to the LPS group.

The Foxp3 mRNA expression was higher in the LPS group compared to the control (*p* = 0.001) and PM+LPS (*p* = 0.001). Moreover, the PM group tended to increase compared to the PM+LPS group (*p* = 0.06). The two-way ANOVA showed an interaction between the LPS nebulization and the PM_2.5_ exposure (*p* ≤ 0.0001) (Figure 3).

### 2.5. Inflammatory Cytokines

Besides the different inflammatory profiles that were analyzed in the blood serum, BALF, and lung parenchyma, we also analyzed the mRNA expression of the TLR-2, TLR-4, and MyD-88. All data from the inflammatory cytokines assessment are presented in Table 2.

The mRNA expression of the TLR-2 was not different among the groups (Figure 3). The mRNA expression of the TLR-4 was higher in the LPS group compared to the control (*p* ≤ 0.0001), PM (*p* ≤ 0.0001), and PM+LPS (*p* ≤ 0.0001) group (Figure 3). Following the same pattern, the mRNA expression of MyD-88 was also higher compared to the control (*p* ≤ 0.0001), PM (*p* ≤ 0.0001), and PM+LPS (*p* ≤ 0.0001) groups (Figure 3). The two-way ANOVA analysis showed that the results from the TLR-4 and MyD-88 assessments were influenced by the LPS nebulization (*p* ≤ 0.0001 for both variables), PM_2.5_ exposure (*p* ≤ 0.0001 and *p* = 0.001, respectively), and by their interaction (*p* ≤ 0.0001 for both variables).

The group LPS had higher levels of KC in the blood serum compared to the control (*p* ≤ 0.0001), PM (*p* ≤ 0.0001), and PM+LPS (*p* = 0.003), these results were influenced by the LPS nebulization (*p* ≤ 0.0001), PM_2.5_ exposure (*p* = 0.007), and their interaction (*p* = 0.013). Moreover, in the BALF, the group LPS also had higher levels of KC compared to the control (*p* ≤ 0.0001), PM (*p* ≤ 0.0001), and PM+LPS (*p* = 0.017) groups. The PM+LPS group had increased KC levels in the BALF compared to the control (*p* ≤ 0.0001) and PM (*p* ≤ 0.0001) groups. The results in the BALF were also influenced by the LPS nebulization (*p* ≤ 0.0001), PM_2.5_ exposure (*p* = 0.03), and their interaction (*p* = 0.018).

**Figure 4:**
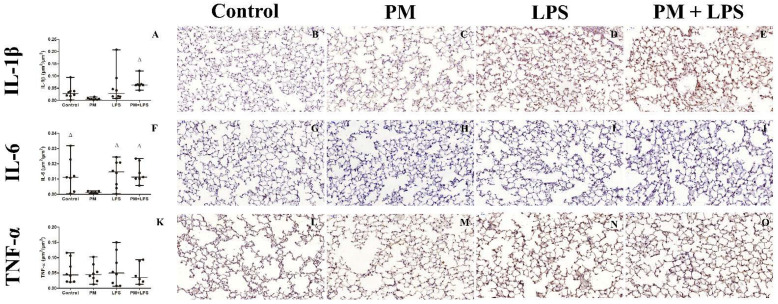
Graphical representation of IL-1β, IL-6, and TNFα assessment in the lung parenchyma and representative photomicrographs of immunostained lung tissue. (**A**) Graphical representation of IL-1β in the lung parenchyma. Representative photomicrographs of the lung parenchyma immunostained with anti-IL-1β antibody: (**B**) control group, (**C**) PM group, (**D**) LPS group, and (**E**) PM+LPS group. The PM+LPS group exhibits intense positive stains for IL-1β mostly in the epithelial cells, while in the LPS group the positive stains for IL-1β are mostly in inflammatory cells. (**F**) Graphical representation of IL-6 in the lung parenchyma. Representative photomicrographs of the lung parenchyma immunostained with anti-IL-6 antibody: (**G**) control group, (**H**) PM group, (**I**) LPS group, and (**J**) PM+LPS group. The LPS group exhibits positive stains for IL-6 mostly in the inflammatory cells and epithelial cells, while in the control and PM+LPS groups the positive stains for IL-6 are mostly in epithelial cells. (**K**) Graphical representation of TNF-α in the lung parenchyma. Representative photomicrographs of the lung parenchyma immunostained with anti-TNFα antibody: (**L**) control group, (**M**) PM group, (**N**) LPS group, and (**O**) PM+LPS group. Positive stains for TNFα are mostly in the epithelial cells. Each point of the graph represents one animal and bars show the median. ∆ *p* < 0.05 compared to the PM group. Scale bars = 50 μm.

There was no difference in the IL-1beta levels detected in the blood serum. In the BALF, the levels of IL-1beta were higher in the LPS group compared to the control (*p* ≤ 0.0001), PM (*p* ≤ 0.0001), and a tendency towards increase compared to the PM+LPS group (*p* = 0.06) was observed. In addition, the levels of IL-1beta were also higher in the PM+LPS group compared to the control (*p* = 0.001) and PM (*p* = 0.002) groups, as a result of the LPS nebulization (*p* ≤ 0.0001). In the lung parenchyma, the proportion of IL-1beta was increased in the PM+LPS group compared to the PM (*p* = 0.026) group, also due to the LPS nebulization (*p* = 0.005) (Figure 4).

The serum levels of IL-6 in the LPS group were higher than in the PM group (*p* = 0.027) and tended to increase compared to the control (*p* = 0.06), influenced only by the LPS nebulization (*p* = 0.002). In the BALF, the LPS group had greater levels of IL-6 compared to the control (*p* ≤ 0.0001), PM (*p* ≤ 0.0001), and PM+LPS (*p* ≤ 0.0001) groups. The PM+LPS also had higher levels of IL-6 in the BALF compared to the control (*p* = 0.008) and PM (*p* = 0.008) groups. The BALF levels of IL-6 were influenced by the LPS nebulization (*p* ≤ 0.0001), PM_2.5_ exposure (*p* ≤ 0.0001), and their interaction (*p* ≤ 0.0001). In the lung parenchyma, the proportion of IL-6 was decreased in the PM group compared to the control (*p* = 0.044), LPS (*p* = 0.03), and PM+LPS (*p* = 0.027), as a result of the LPS nebulization (*p* = 0.035) and the interaction between LPS nebulization and PM_2.5_ exposure (*p* = 0.044) (Figure 4).

The total TNF levels were increased in the LPS group compared to the control (*p* = 0.001), PM (*p* = 0.001), and PM+LPS (*p* = 0.009) groups in the blood serum, as a consequence of the LPS nebulization (*p* = 0.001) and the interaction between LPS nebulization and PM_2.5_ exposure (*p* = 0.009). Following the same pattern, the BALF total TNF levels were higher in the LPS group compared to the control (*p* ≤ 0.0001), PM (*p* ≤ 0.0001), and PM+LPS (*p* ≤ 0.0001), as a result of the LPS nebulization (*p* ≤ 0.0001), PM_2.5_ exposure (*p* ≤ 0.0001), and their interaction (*p* ≤ 0.0001). In the lung parenchyma, the proportion of TNF-alpha had no significant difference among the groups (Figure 4).

**Figure 5:**
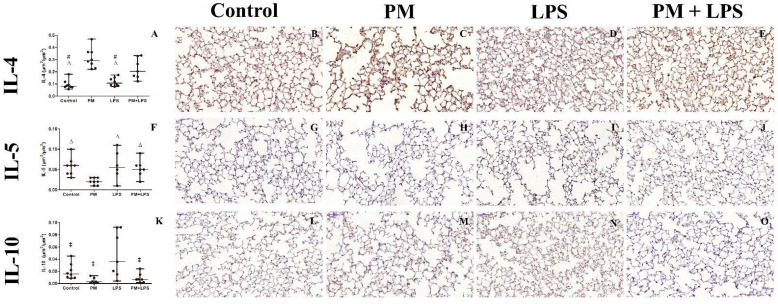
Graphical representation of IL-4, IL-5, and IL-10 assessment in the lung parenchyma and representative photomicrographs of immunostained lung tissue. (**A**) Graphical representation of IL-4 in the lung parenchyma. Representative photomicrographs of the lung parenchyma immunostained with anti-IL-4 antibody: (**B**) control group, (**C**) PM group, (**D**) LPS group, and (**E**) PM+LPS group. Positive stains for IL-4 are mostly in the epithelial cells. (**F**) Graphical representation of IL-5 in the lung parenchyma. Representative photomicrographs of the lung parenchyma immunostained with anti-IL-5 antibody: (**G**) control group, (**H**) PM group, (**I**) LPS group, and (**J**) PM+LPS group. Positive stains for IL-5 are mostly in the epithelial cells. (**K**) Graphical representation of IL-10 in the lung parenchyma. Representative photomicrographs of the lung parenchyma immunostained with anti-IL-10 antibody: (**L**) control group, (**M**) PM group, (**N**) LPS group, and (**O**) PM+LPS group. In the LPS group, positive stains for IL-10 are mostly in the inflammatory cells. Each point of the graph represents one animal and bars show the median. ^‡^ *p* < 0.05 compared to the LPS group. ∆ *p* < 0.05 compared to the PM group. # *p* < 0.05 compared to the PM+LPS group. Scale bars = 50 μm.

The IL-4 levels in the blood serum and BALF had no difference among the groups. In the lung parenchyma, the PM+LPS group had increased IL-4 compared to the control (*p* = 0.003) and LPS (*p* = 0.013) groups. The PM group had even higher levels of IL-4 compared to the control (*p* ≤ 0.0001), LPS (*p* ≤ 0.0001), and tended to increase compared to the PM+LPS (*p* = 0.06) group. These results were influenced by the PM_2.5_ exposure (*p* ≤ 0.0001) and by the interaction between LPS nebulization and PM_2.5_ exposure (*p* = 0.027) (Figure 5).

The blood serum IL-5 levels were lower in the LPS (*p* = 0.004) and PM+LPS (*p* = 0.003) groups compared to the PM group, influenced by the LPS nebulization (*p* ≤ 0.0001). The levels of IL-5 were below the limit of detection in the BALF samples. In the lung parenchyma, the levels of IL-5 were lower in the PM group compared to the control (*p* = 0.02) and LPS (*p* = 0.018) groups, due to the PM_2.5_ exposure (*p* = 0.015) (Figure 5).

The levels of IL-10 in the blood serum and BALF were not statistically different among the groups. In the lung parenchyma, the IL-10 was increased in the LPS group compared to the PM (*p* = 0.003) and PM+LPS (*p* = 0.008) groups, as a result of the LPS nebulization (*p* = 0.04) and PM_2.5_ exposure (*p* = 0.001) (Figure 5).

**Figure 6:**
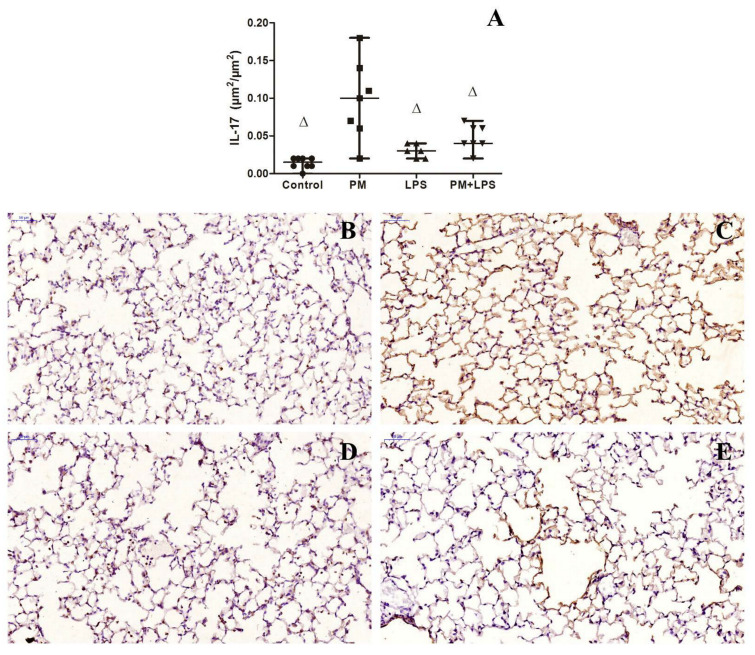
Graphical representation of IL-17 assessment in the lung parenchyma and representative photomicrographs of immunostained lung tissue. (**A**) Graphical representation of IL-17 in the lung parenchyma. Representative photomicrographs of the lung parenchyma immunostained with anti-IL-17 antibody: (**B**) control group, (**C**) PM group, (**D**) LPS group, and (**E**) PM+LPS group. The IL-17 staining has a different pattern in each group. The PM group exhibits intense positive stains for IL-17 mostly in the epithelial cells. The LPS group exhibits positive stains for IL-17 mostly in the inflammatory. The PM+LPS group also exhibits intense positive stains for IL-17 mostly in the epithelial cells, however with a very irregular distribution, staining mainly the alveolar ducts. Each point of the graph represents one animal and bars show the median. ∆ *p* < 0.05 compared to the PM group. Scale bars = 50 μm.

There was no statistical difference in the blood serum IL-17 levels among the groups. The LPS groups had higher levels of IL-17 in the BALF compared to the control (*p* = 0.022) and PM (*p* = 0.02) groups, as a result of the LPS nebulization (*p* = 0.009). In the lung parenchyma, the PM group had higher levels of IL-17 compared to the control (*p* ≤ 0.0001), LPS (*p* = 0.001), and PM+LPS (*p* = 0.014) groups. The PM+LPS tented to increase compared to the control group (*p* = 0.06). The IL-17 levels in the lung parenchyma were influenced by PM_2.5_ exposure (*p* ≤ 0.0001) and by the interaction between LPS nebulization and PM_2.5_ exposure (*p* = 0.004) (Figure 6).

## 3. Discussion

Exposure to air pollution is a risk factor for respiratory and cardiovascular non-communicable diseases and the fine particulate matter, which can reach the deepest parts of the lungs, can regulate several inflammatory pathways, cytokines, and genes leading to an inflammation-related injury [1,6]. Our study suggests that sub-chronic exposure to fine particulate matter can alter the inflammatory response induced by LPS. Like the LPS group, the PM+LPS group showed histological evidence of injury and septal thickening, increased BALF leukocytes and neutrophils, increased macrophages in the lung tissue, increased levels of KC and IL-6 in the BALF, and increased levels of IL-1β in the BALF and lung parenchyma. However, unlike the LPS group, it did not show changes in MPO+ neutrophils and CD3+ lymphocytes in the lung parenchyma, in the levels of TNF or IL-10, or in the expression of TLR-4 and MyD-88. Moreover, like the PM group, the PM+LPS group had increased levels of IL-4 and IL-17 in the lung parenchyma, but unlike the PM group, it did not show changes in the expression of FOXP3 in the lung tissue. It is worth noting that, although not always statistically different, all increased cytokines in the PM+LPS group (KC, IL-6, IL-1β, IL-4, and IL-17) are not as increased as in the reference group (LPS or PM group).

LPS is a potent microbial inducer of inflammation and its inflammatory response in the lungs is characterized by the polymorphonuclear influx, high levels of MPO, and increased pro-inflammatory cytokines in the BALF [20]. The LPS group showed the expected inflammatory response to the LPS exposure. Besides the histological evidence of tissue injury and septal thickening, the LPS group also showed increased BALF leukocytes, increased neutrophils in the blood, BALF, and lung parenchyma, and increased lung tissue T-lymphocytes. Furthermore, the LPS group also showed higher levels of the pro-inflammatory cytokines KC, IL-6, and TNF in blood serum and BALF, increased IL-1 beta and IL-17 in the BALF, and increased mRNA expression of FOXP3, TLR4, and MyD88.

The role of the TLR-4 and MyD88 in LPS-induced inflammation is well described in the literature [21]. Our results showed that the LPS group had increased mRNA expression of TLR-4 and MyD-88, whereas the group PM+LPS did not display this pattern. Even though we did not measure the protein expression of TLR-4 and MyD-88, the changes in their mRNA expression may indicate that they may play a role in the process.

The recognition of LPS by the immune system is an important first step in identifying invading pathogens and in initiating a protective immune response; therefore, any impairment in this process can potentially increase the susceptibility to certain infectious agents. It has been reported that exposure to air pollution increases the susceptibility to infections caused by bacteria, such as *Pseudomonas aeruginosa* [22], *Streptococcus pneumoniae* [23], and *Mycobacterium tuberculosis* [24]. In addition, there is also an association between exposure to air pollution and the transmission/severity of viral infections [25].

The PM group displayed a very different inflammatory pattern from the LPS group, with the increase in circulating leukocytes and lymphocytes, increased BALF and lung parenchyma macrophages, increased levels of IL-5 in the blood serum, and increased levels of IL-4 and IL-17 in the lung parenchyma. Many studies have shown the connection between the concentration of PM_2.5_ and the development or exacerbation of allergies and inflammatory/autoimmune disorders [7].

Previous studies suggest that exposure to PM_2.5_ can impair the macrophages’ phagocytosis [26] and pathogen opsonization, decrease antigen presentation [27,28], and disturb the Th1/Th2 balance, switching to a Th2-dominant profile, mainly via the presence of PAHs in the PM_2.5_ [6]. In addition, when relatively low levels of LPS are inhaled, a cascade of immune responses leads to Th2 cells differentiation, activation of dendritic cells, and the increase in IL-4, IL-5, and IL-13 [29].

IL-17A is a cytokine that acts as part of the host defense to bacterial and fungal infections and aberrant IL-17 signaling can lead to excess inflammation [30]. Previous studies showed that exposure to air pollution increases the levels of IL-17 and enhances Th17 differentiation through the aryl hydrocarbon receptor (AHR) [31,32]. Moreover, Gałuszka et al. [7] reported that T cells exposed to transition metal-containing particulate matter showed an increase in the proportion of IFN-γ and IL-17A producing cells, with a concomitant decrease in Treg cells. Our results suggest a Th17/Treg imbalance in our animal exposed to PM_2.5_ with increased expression of IL-17 in the lung parenchyma and decreased expression of Foxp3 mRNA. Although the Foxp3 mRNA expression may not exclusively represent Tregs, there is evidence that mRNA expression and immunostained Foxp3 cells are correlated [33]. In addition, Nadeau et al. [34] showed that PM_2.5_ suppresses Treg through its ability in causing hypermethylation of the gene encoding the Treg-transcription factor (Foxp3) in the peripheral blood of children with asthma.

The organic compounds present in the PM_2.5_, such as pollen, fungal spores, mold, and microbial components (including LPS) also play an important role in inflammation modulation [6] and, we have confirmed the presence of LPS in the PM_2.5_ that the animals were exposed [19]. Nomura et al. [35] showed that macrophages exposed to low levels of LPS show reduced response to a second stimulation with LPS by downregulating TLR4 leading to a time- and dose-dependent reduction in the production of inflammatory cytokines. This LPS tolerance compromises the ability of macrophages to mount an effective immune response during a subsequent pathogen encounter [36]. A growing body of evidence suggests that the effects of air pollution exposure on the pathogenesis of respiratory infections may be pollutant-, timing-, and infection-specific [37].

Air pollution is a great environmental risk to health and PM_2.5_ exposure is the fifth leading risk factor for death in the world, accounting for 4.2 million deaths [1]. The Metropolitan Area of São Paulo is a megacity with a population of 21 million, corresponding to more than 11% of the total population of Brazil [38]. The São Paulo State (Brazil) air quality standards recommend that the 24 h mean PM_2.5_ concentration should not exceed 60 μg·m^−3^ [39], which is fourfold higher than the new 24 h mean level of 15 μg·m^−3^ recommended by the World Health Organization [2]. According to the São Paulo State air quality standard classification, the 24 h mean of 50.8 μg·m^−3^ would be in the range between moderate and bad air quality. From 2016 to 2020, the annual percentage distribution of days that had moderate air quality or worse in the monitoring stations ranged from 13.8 to 16.3% [39].

Research on PM_2.5_ and the interpretation of research findings on exposure and risk are complicated by this heterogeneity and the possibility that the potential of particles to cause injury varies with size and other physical characteristics, chemical composition, and source(s) [2].

All the employed methodology in this study was selected taking into account the availability of our materials and resources. One of the strengths of our study is the PM_2.5_ exposure method. The ambient particle concentrator mimics human whole-body exposure, concentrating the “real world” particles with no need of sedation and minimum impact on mice’s food intake and drinking during the exposure. Animal models are an important tool to test hypotheses offering controlled conditions and generating reproducible results; however, several limitations should be considered when investigating the effects of inhaled PM_2.5_. There are considerable differences between the human and mouse respiratory system anatomy and immunology. To cite a few examples: mice have complex nasal anatomy, a lower number of cilia, fewer club cells, differences in pulmonary lobulation, bronchial branching, and a lack of respiratory bronchioles, all features that can affect particle retention and distribution [40]. In addition, mice differ from humans regarding the balance of leukocyte subsets, Ig subsets, and chemokine and its receptor expression [41]. Even though the translation of the results into humans may be limited, they bring interesting insights for future studies in humans. Furthermore, it is unclear how the different PM_2.5_ components are responsible for the observed effects.

## 4. Materials and Methods

The Institutional Review Board for Ethics on Animal Use approved all ethics aspects of this project (protocol no. 177/10), including animal manipulation and euthanasia. Moreover, according to the institutional guidelines for animal welfare, all animals were treated with due consideration to the alleviation of distress and discomfort.

### 4.1. Study Design

We designed this experimental study to investigate the effects of exposure to concentrated PM_2.5_ on LPS-induced lung injury onset. To do so, sixty-four BALB/c male mice (9 weeks old) were obtained from our university’s animal facility and maintained at 22–26 °C and 55–75% humidity under a 12/12 h dark/light cycle with food and water provided ad libitum.

Animals were exposed to ambient PM_2.5_ in an ambient particle concentrator (APC), described in Sioutas et al. [42], developed at the Harvard School of Public Health. The APC is located close to a high-traffic road (23°33′18.1″ S 46°40′15.0″ W), inside the School of Medicine of São Paulo University campus. Animals were placed inside the exposure chambers with controlled temperature, humidity, and pressure, either connected to the concentrate PM_2.5_ stream (PM and PM+LPS groups) or to a clean air supply provided using a high-efficiency particulate air filter (control and LPS groups). The mass concentrations of PM_2.5_ were measured using an airborne particulate monitor (two-wavelength nephelometer, DataRam DR-4000, Thermo Fisher Scientific, Waltham, MA, USA) during the exposure. During the exposure, the concentration of PM_2.5_ is susceptible to fluctuations; therefore, the time of exposure was adjusted proportionally to the APC-derived PM_2.5_ concentration at the start of each exposure to achieve our target dose of 1200 µg·m^−3^ for 1 h daily, not exceeding the limit of 120 min per day.

The acute lung injury was induced with nebulized LPS (Lipopolysaccharides from Escherichia coli O111:B4—Sigma-Aldrich, St. Louis, MO, USA) at a 3 mg/mL concentration as previously described by Costa et al. [43].

Therefore, four experimental groups were established: 1. Control group—mice exposed to filtered air for 5 weeks and exposed to nebulized saline. 2. PM group—mice exposed to PM_2.5_ (daily dose: 1200 µg·m^−3^) for 5 weeks and exposed to nebulized saline. 3. LPS group—mice exposed to filtered air for 5 weeks and exposed to nebulized LPS. 4. PM+LPS—mice exposed to PM_2.5_ (daily dose: 1200 µg·m^−3^) for 5 weeks and exposed to nebulized LPS.

All animals were euthanized with an intraperitoneal injection of sodium thiopental (200 mg·kg^−1^ body weight) 24 h after the nebulization to either LPS or saline. Blood samples were collected from all animals. From 7–8 animals of each group, samples from one lung were frozen for the molecular analysis and the other lung fixed in 4% buffered paraformaldehyde solution for the histopathological/immunohistochemical evaluation. From the remaining animals (*n* = 7–8 per group), BALF was collected, and both lungs were fixed in 4% buffered paraformaldehyde solution for the stereological analyses.

### 4.2. PM_2.5_ Elementary Characterization

PM_2.5_ was collected in polycarbonate filter membranes. Concentrations of metal trace elements and black carbon (BC) concentration were assessed as previously described by Andrade et al. [15] and de Miranda et al. [16]. The polycyclic aromatic hydrocarbon (PAHs) content of PM_2.5_ was assessed as previously described by Yoshizaki et al. [18]. The endotoxin content in the PM_2.5_ was quantified with the ToxinSensor Chromogenic LAL Endotoxin Assay Kit (GenScript, Piscataway, NJ, USA), as recommended by the manufacturer’s instructions.

### 4.3. Blood and Bronchoalveolar Lavage Fluid (BALF) Analysis

Full and differential cell counts were performed in the total blood and BALF samples. Differential cell counts were performed using the May-Grünwald-Giemsa stain (300 cells per animal). Inflammatory cytokines IL-1β, IL-4, IL-5, IL-6, IL-10, IL-17, KC, and total TNF were quantified by the cytometric bead assay (BD Bioscience, San Jose, CA, USA) according to the manufacturer’s instructions, in the BALF and blood serum. One thousand and two hundred events were acquired by a BD FACSCanto II flow cytometer (BD Biosciences, San Jose, CA, USA), and data were analyzed with FCAP Array software (BD Biosciences, San Jose, CA, USA).

### 4.4. Stereological Analysis and Descriptive Analysis

Lung stereology was carried out as described in Hsia et al. [44] using newCAST software (Visiopharm, Hørsholm, Denmark). Briefly, lungs were sampled, fixed in 4% buffered paraformaldehyde solution, and paraffin-embedded. Five-micrometer-thick sections were stained with hematoxylin and eosin (H&E) for lung structure evaluation. The total lung volume and its compartments, and volume densities were estimated by the Cavaliere principle. The density surface, the total surface area of the alveolar septa, and the arithmetic mean thickness were also assessed as described in Hsia et al. [44].

In addition, a semi-quantitative analysis of the inflammation was performed by an experienced pathologist to determine histopathological characteristics using the following scores: grade 0 (absent), 1 (discrete), 2 (mild), 3 (moderate), and 4 (intense) [45].

### 4.5. Molecular Analysis

Toll-like Receptor (TLR)-2, TLR4, MyD-88, and Foxp3 mRNA were quantified by real-time PCR using specific primers. All primer sequences are presented in Supplementary Table S2.

Total RNA of the frozen lungs was extracted with TRIzol (Ambion, Life Technologies, Carlsbad, CA, USA), cDNA synthesis (SuperScript VILO cDNA Synthesis Kit, Invitrogen, Life Technologies, Carlsbad, CA, USA), and real-time PCR (Fast SYBR Green Master Mix, Applied Biosystems, Life Technologies, Carlsbad, CA, USA) were conducted according to the manufacturer’s protocols. The relative expression of the transcripts was calculated after normalization to the levels of the reference gene of the ribosomal protein L13A [46].

### 4.6. Immunohistochemically Inflammation Assessment

Lung tissue was immunostained using anti-IL-1β, anti-IL-4, anti-IL-5, anti-IL-6, anti-IL-10, anti-IL-17, and anti-TNF-α antibodies. The inflammatory cytokines were quantified in the lung parenchyma by measuring the proportional area (stained area/lung tissue area) in 15 HPFs per animal using the Image-Pro Plus 4.1 software (Media Cybernetics, Silver Spring, MD, USA). Furthermore, lung tissue sections were immunostained using anti-myeloperoxidase (MPO), anti-CD3, and anti-MAC2 antibodies. The immunostained cells were counted in 20 high power fields (HPFs) and the proportion of cells per area of lung tissue was calculated. The standardized dilutions of the antibodies and their commercial sources are presented in Supplementary Table S3.

### 4.7. Statistical Analysis

We used the SPSS 21 software (SPSS Inc/IBM, Chicago, IL, USA) for the statistical analyses and the GraphPad Prism 7 (GraphPad Software, La Jolla, CA, USA) for data visualization. Descriptive statistics were calculated for each variable and group. We performed ANOVA or the Kruskal–Wallis test, followed by Tukey or Bonferroni post hoc tests to compare the groups according to the normality of distribution, previously assessed by the Kolmogorov–Smirnov normality test. Furthermore, we performed a two-way analysis of variance (two-way ANOVA) to verify the influence of the LPS nebulization, PM_2.5_ exposure, and their interaction on each variable. Statistical differences were assumed at the 5% significance level.

## 5. Conclusions

PM_2.5_ is a heterogeneous mixture of organic and inorganic components and, even with all the findings highlighting its complexity and dynamic nature, its effects in the lung are not fully understood. Their ability to cause injury may vary with their size, physical and chemical characteristics, and source. Different characteristics of PM_2.5_ may be relevant to different health effects. The animals with LPS-induced acute lung injury that were previously exposed to PM_2.5_ showed a mixed inflammatory response, and our analysis shows that there is often an interaction between the LPS nebulization and PM_2.5_ exposure, modulating differently the inflammatory response, with a distinct pattern when compared with the LPS or the PM_2.5_ exposure alone.

Further studies are required to explain the mechanisms of immune modulation caused by chronic exposure to PM_2.5_ and how it could alter the onset, progression, and resolution of acute lung injury and the susceptibility to secondary respiratory infections.

## Figures and Tables

**Table 1:** Semi-quantitative analysis of the inflammation and stereological assessment of the lung tissue.

Parameter	Control	PM	LPS	PM+LPS	LPS Nebulization	PM_2.5_ Exposure	Interaction
Initial Body Weight (g)	22 (3)	23 (2)	22 (2)	24 (4)	ns	ns	ns
Final Body weight (g)	24 (2) ^bc^	26.5 (3.5) ^bc^	21.5 (2)	20 (4)	*p* ≤ 0.0001	ns	*p* = 0.042
Peribronchial inflammation score	0 (1) ^bc^	1 (1) ^bc^	1.5 (1)	1.5 (1)	*p* ≤ 0.0001	ns	ns
Alveolar inflammatory infiltrate score	0 (1) ^b^	0 (1) ^b^	2 (1)	1 (1)	*p* ≤ 0.0001	ns	ns
Lung volume (mm^3^)	431.2 (145)	486.1 (172)	462.6 (55)	470.4 (94)	ns	ns	ns
Lung volume per body weight ratio (mm^3^/g)	18.8 (4.7)	17.5 (8.4)	20.9 (3.3)	22.7 (3) ^a^	*p* = 0.02	ns	ns
Vv parenchyma (%)	78.1 (10)	78.5 (6)	81.6 (8)	77.6 (8)	ns	ns	ns
Vv septa (%)	28.8 (7.4)	32.8 (4.7)	34.3 (3.3)	34.5 (7.7) ^a^	*p* = 0.031	ns	ns
Vv alveolar airspace (%)	49.3 (2.6)	46.9 (5.8) ^c^	48.6 (6.1)	44.3 (5.8)	ns	*p* = 0.01	ns
Vv non-parenchyma (%)	21.9 (15)	21.5 (6)	18.4 (8)	22.4 (8)	ns	ns	ns
Vt parenchyma (mm^3^)	332.9 (116.8)	392.2 (162.6)	368.4 (48.8)	369.2 (50.9)	ns	ns	ns
Vt septa (mm^3^)	125.4 (31.2)	163.5 (61.1) ^a^	181.7 (35.2)	129.9 (70.9)	ns	ns	*p* = 0.002
Vt alveolar airspace (mm^3^)	199.4 (73.9)	230.2 (110) ^c^	264.9 (71.7)	183.6 (63.2)	ns	ns	*p* = 0.004
Vt non-parenchyma (mm^3^)	80.4 (65.5)	105.9 (47.9)	76.5 (40.15)	102 (41.3)	ns	ns	ns
Sv septa (mm^−1^)	238.9 (53.6)	211 (47.1)	199.8 (27.8) ^a^	204.1 (16.9) ^a^	*p* = 0.01	ns	ns
St septa (10^3^ mm^2^)	94.6 (56.9)	113.1 (52)	89.8 (23.9)	92.8 (23.6)	ns	ns	ns
Septal thickness (µm)	2.6 (1)	3.1 (1.2)	3.7 (1.3) ^a^	3.3 (1.7) ^a^	*p* = 0.004	ns	ns

Data are expressed in median (interquartile range). ns: not significant. ^a^ *p* > 0.05 compared to the control group. ^b^ *p* > 0.05 compared to the LPS group. ^c^ *p* > 0.05 compared to the PM+LPS.

**Table 2:** Inflammatory cytokines.

	Control	PM	LPS	PM+LPS	LPS Nebulization	PM_2.5_ Exposure	Interaction
**KC**							
Serum (pg/mL)	3.8 (1.3) ^b^	3.7 (2.4) ^b^	16.8 (9.7)	7.5 (3) ^b^	*p* ≤ 0.0001	*p* = 0.007	*p* = 0.013
BALF (pg/mL)	0.6 (2.5) ^bd^	0.9 (5.7) ^bd^	103.6 (24.3)	60.7 (74.2) ^b^	*p* ≤ 0.0001	*p* = 0.03	*p* = 0.018
**IL-1** **β**							
Serum (pg/mL)	>LD	>LD	0 (0.04)	>LD	ns	ns	ns
BALF (pg/mL)	>LD ^bc^	>LD ^bd^	23.7 (15.1)	12.9 (7.5)	*p* ≤ 0.0001	ns	ns
Tissue (µm^2^/µm^2^)	0.02 (0.02)	0.005 (0.005) ^d^	0.03 (0.06)	0.06 (0.006)	*p* = 0.005	ns	ns
**IL-6**							
Serum (pg/mL)	0.1 (0.3)	0.1 (0.2) ^b^	9.3 (7.9)	7.7 (13.2)	*p* = 0.002	ns	ns
BALF (pg/mL)	>LD ^bd^	0.01 (0.3) ^bd^	800.8 (613.5)	325.3 (231.2) ^b^	*p* ≤ 0.0001	*p* ≤ 0.0001	*p* ≤ 0.0001
Tissue (µm^2^/µm^2^)	0.01 (0.02) ^c^	0.001 (0.002)	0.02 (0.02) ^c^	0.01 (0.01) ^c^	*p* = 0.035	ns	*p* = 0.044
**TNF**							
Serum (pg/mL)	0.8 (1.2) ^b^	0.9 (1.7) ^b^	5.1 (3.4)	2.3 (0.8) ^b^	*p* = 0.001	ns	*p* = 0.009
BALF (pg/mL)	0.01 (0.4) ^b^	0.1 (1) ^b^	2822.1 (1310.3)	486.5 (379.8) ^b^	*p* ≤ 0.0001	*p* ≤ 0.0001	*p* ≤ 0.0001
Tissue (µm^2^/µm^2^)	0.04 (0.07)	0.04 (0.05)	0.05 (0.1)	0.03 (0.07)	ns	ns	ns
**IL-4**							
Serum (pg/mL)	>LD	>LD	>LD	>LD	ns	ns	ns
BALF (pg/mL)	>LD	>LD	>LD	>LD	ns	ns	ns
Tissue (µm^2^/µm^2^)	0.08 (0.03) ^cd^	0.3 (0.1)	0.1 (0.06) ^cd^	0.2 (0.2)	ns	*p* ≤ 0.0001	*p* = 0.027
**IL-5**							
Serum (pg/mL)	0.3 (0.5)	0.8 (0.8)	>LD ^c^	>LD ^c^	*p* ≤ 0.0001	ns	ns
BALF (pg/mL)	>LD	>LD	>LD	>LD	ns	ns	ns
Tissue (µm^2^/µm^2^)	0.05 (0.06) ^c^	0.02 (0.01)	0.06 (0.06) ^c^	0.05 (0.02)	ns	*p* = 0.015	ns
**IL-10**							
Serum (pg/mL)	0.4 (1.5)	0.1 (0.7)	1.1 (2.3)	0 (0.1)	ns	ns	ns
BALF (pg/mL)	0 (0.9)	0 (0.3)	1.1 (2.9)	0 (0.4)	ns	ns	ns
Tissue (µm^2^/µm^2^)	0.015 (0.02)	0.003 (0.008) ^b^	0.035 (0.08)	0.007 (0.01) ^b^	*p* = 0.04	*p* = 0.001	ns
**IL-17A**							
Serum (pg/mL)	1.7 (0.4)	1.8 (0.4)	1.5 (0.5)	1.6 (0.1)	ns	ns	ns
BALF (pg/mL)	1.2 (0.1) ^b^	1.2 (0.2) ^b^	6.5 (9.3)	3.9 (3.1)	*p* = 0.009	ns	ns
Tissue (µm^2^/µm^2^)	0.01 (0.01) ^c^	0.1 (0.08) ^c^	0.03 (0.01)	0.04 (0.03) ^c^	ns	*p* ≤ 0.0001	*p* = 0.04

Data are expressed in median (interquartile range). >LD, below the limit of detection. ns, not significant. **^a^** *p* > 0.05 compared to the control group. ^b^ *p* > 0.05 compared to the LPS group. ^c^ *p* > 0.05 compared to the PM. ^d^ *p* > 0.05 compared to the PM+LPS.

## Data Availability

All relevant data are within the paper and its Supporting Information Files.

## Supplementary Materials

The following supporting information can be downloaded at: https://www.mdpi.com/article/10.3390/ijms23073913/s1.
