# Supplementary material for: LPS Response Is Impaired by Urban Fine Particulate Matter
Source: Int J Mol Sci. 2022 Apr 1;23(7):3913. doi: 10.3390/ijms23073913 (PMC8998903; doi:10.3390/ijms23073913)
Supplement: Supplementary file 1 [file ijms-23-03913-s001.zip › ijms-1586777-supplementary.pdf]

Supplementary Material

LPS response is impaired by urban fine particulate matter

Natália de Souza Xavier Costa [1], Gabriel Ribeiro Júnior [1], Adair Aparecida dos Santos Alemany [1], Luciano Belotti [1], Marcela Frota Cavalcante [2], Susan Ribeiro [3,4], Mariana Matera Veras [1], Esper Georges Kallás [3], Paulo Hilário Nascimento Saldiva [1], Marisa Dolhnikoff [1], Luiz Fernando Ferraz da Silva [1]

[1] Departamento de Patologia, LIM-05, Faculdade de Medicina da Universidade de São Paulo, Avenida Dr. Arnaldo, 455, São Paulo, São Paulo 01246-903, Brazil.

[2] Departamento de Análises Clínicas E Toxicológicas, Faculdade de Ciências Farmacêuticas da, Universidade de São Paulo, São Paulo, São Paulo, Brazil.

[3] Laboratório de Imunologia Clínica E Alergia (LIM60), Faculdade de Medicina da Universidade de São Paulo, São Paulo, São Paulo, Brazil.

[4] Department of Pathology, Case Western Reserve University, Cleveland, OH, USA.

*Email: nataliasxcosta@usp.br

Table S1: Red blood cell count, fibrinogen (mg/dL) and platelet (1000/mm³) quantification.

	Control	PM	LPS	PM+LPS	LPS nebulization	PM2.5 exposure	Interaction
**Erythrocytes (millions/mm³)**	9.57 (1.02)	8.8 (0.88) [b]	10.0 (0.46)	8.87 (0.82) [b]	ns	$p \leq 0.0001$	$p = 0.018$
**Hemoglobin (g/dL)**	15.8 (1.5)	14.5 (1.5) [b]	16.8 (1.0)	15.1 (1.7) [b]	ns	$p = 0.001$	ns
**Mean corpuscular volume – MCV (fL)**	48.15 (0)	47.3 (1) [c]	48.15 (1)	50 (1)	$p = 0.009$	ns	$p = 0.008$
**Mean corpuscular hemoglobin – MCH (pg)**	16.5 (0.3)	16.3 (0.4)	16.45 (0.6)	17.3 (0.4)	ns	ns	ns
**Mean corpuscular hemoglobin concentration – MCHC(g/dL)**	34.3 (0.5)	34.6 (0.8)	34.2 (0.9)	34.6 (0.3)	ns	ns	ns
**Red Cell Distribution Width (%)**	15.25 (0)	15.4 (1)	15.55 (1)	16.3 (1)	ns	ns	ns
**Hematocrit (%)**	46.1 (4.9)	42.2 (4.1) [b]	18.7 (1.8)	43.7 (3.1) [b]	ns	$p = 0.001$	ns
**Fibrinogen (mg/dL)**	285 (70)	155 (70) [a]	390 (0) [ab]	190 (0) [ab]	$p \leq 0.0001$	$p \leq 0.0001$	$p = 0.023$
**Platelets (1000/mm³)**	1198 (192)	787 (362) [ab]	1334 (333)	1000 (269) [ab]	ns	$p \leq 0.0001$	ns

Data are expressed in median (interquartile range). ns: not significant. [a] $p > 0.05$ compared to the control group. [b] $p > 0.05$ compared to the LPS group. [c] $p > 0.05$ compared to the PM+LPS.

Table S2: Primer Sequences

Gene	Forward Sequence	Reverse Sequence
**RPL13a**	AACCTTTGGTCCCCACTTCCCT	TCCTCAAGACCAACGGACTCCT
**TLR- 2**	CAGCTGGAGAACTCTGACCC	CAAAGAGCCTGAAGTGGGAG
**TLR- 4**	TCATGGCACTGTTCTTCTCCT	CATCAGGGACTTTGCTGAGTT
**MyD88**	CACCTGTGTCTGGTCCATTG	AGGCTGAGTGCAAACTTGGT
**FOXp3**	TTCATGCATCAGCTCTCCAC	CTGGACACCCATTCCAGACT

RPL13a: Ribosomal Protein L13a. TLR: Toll-Like Receptor. MyD88: Myeloid differentiation primary response 88. FOXp3: Forkhead box P3.

Table S3: Immunohistochemistry standards

	Antibody	Concentration
**Neutrophils**	Anti – MPO (Abcam – cod. ab9535)	1:750
**Lymphocytes**	Anti - CD3 (Serotec – cod. MCA1477)	1:800
**Macrophages**	Anti - MAC 2 (Cedarlane – cod. CL8942AP)	1:100,000
**IL-1 beta**	Anti – IL-1beta (Santa Cruz – sc-7887)	1:400
**IL-4**	Anti – IL-4 (Santa Cruz – sc-1260)	1:1000
**IL-5**	Anti – IL-5 (Santa Cruz – sc-1265)	1:1000
**IL-6**	Anti – IL-6 (Santa Cruz – sc-1265)	1:1500
**IL-10**	Anti – IL-10 (Santa Cruz - sc-73309)	1:100
**IL-17**	Anti – IL-17 (Santa Cruz – sc-6077)	1:2000
**TNF alpha**	Anti – TNF-alpha (Santa Cruz –sc-1348)	1:3000

CD3: Cluster of differentiation 3. IL: Interleukin. MPO: Myeloperoxidase. TNF: Tumor necrosis factor.
